# Emodin Interferes With Nitroglycerin-Induced Migraine in Rats Through CGMP-PKG Pathway

**DOI:** 10.3389/fphar.2021.758026

**Published:** 2021-10-20

**Authors:** Shuding Sun, Guo Zheng, Decui Zhou, Lili Zhu, Xin He, Chunfeng Zhang, Chongzhi Wang, Chunsu Yuan

**Affiliations:** ^1^ School of Traditional Chinese Pharmacy, China Pharmaceutical University, Nanjing, China; ^2^ School of Biomedical Sciences, Faculty of Medicine, The Chinese University of Hong Kong, Hong Kong, Hong Kong, SAR China; ^3^ Tang Center of Herbal Medicine Research and Department of Anesthesia and Critical Care, University of Chicago, Chicago, IL, United States

**Keywords:** emodin, anti-migraine, CGRP, c-fos, cGMP-PKG pathway

## Abstract

The purpose of this research was to explore the effect and mechanism of emodin in interfering with nitroglycerin-induced migraine rats. We carried out behavioral research within 2 h post-nitroglycerin (NTG) injection, and blood samples were collected through the abdominal aorta for measurements of nitric oxide (NO), calcitonin gene-related peptide (CGRP), substance P (SP), tumor necrosis factor (TNF-α) and cyclic guanosine monophosphate (cGMP) levels. Immunohistochemistry was adopted to detect the activation of c-Fos immunoreactive neurons in brain tissues. The number and integrated optical density (IOD) of c-Fos positive cells were measured using Image-Pro Plus. Western blotting was applied to detect the levels of PKG protein in rat brain tissues. The results showed that emodin can alleviate the pain response of migraine rats and significantly reduce the levels of NO, CGRP, SP, TNF-α and cGMP in migraine rats. In addition, emodin can significantly reduce the number of c-Fos positive cells and the IOD value. Moreover, the expression of PKG protein was significantly inhibited by emodin. Therefore, it is inferred that emodin can relieve migraine induced by NTG through the cGMP-PKG pathway, and can be used as a potential botanical medicine for the treatment of migraine.

## Introduction

Migraine is the most common neurovascular disease. It is manifested as severe blood vessel throbbing pain and headache recurring. Patients with migraine are usually sensitive to sound and light, and are generally accompanied with dizziness, vomiting, and nausea (Rajapakse and Davenport 2019) (Vries et al., 2020). The 1-year period prevalence of migraine globally is 15%, and the number of patients worldwide exceeds 1 billion. It ranks second among the world’s disabling diseases and first among women (Steiner et al., 2020). Migraine patients will be greatly affected in their work and life, which will lead to depression, anxiety and other mental illnesses, even self-harm, and a serious decline in the quality of life (Ashina et al., 2021).

There are many hypotheses about the mechanism of migraine, containing vascular hypothesis, neurology, trigeminal neurovascular hypothesis, ion hypothesis and genetic hypothesis. The current mainstream theory is the trigeminal neurovascular theory, which believes that a series of stimulus (mechanical, electrical or chemical, chronic stress, diet, hormonal fluctuations, or events like cortical spreading depression, etc.) acting on intracranial receptors (dural blood vessels, arachnoid and cerebral artery receptors, etc.) can generate a state of “sterile inflammation” resulting in the sensitization and activation of trigeminal meningeal nociceptors, which is the basis for migraine attacking (Zhang et al., 2010; Ramachandran 2018). After the trigeminal nerve endings around the cerebral vessels are stimulated, certain neuropeptides such as CGRP, substance P, neurokinin A (NKA), β-EP, etc. are released, which causes the vascular wall of adjacent intracranial blood vessels to over-dilate, resulting in fluctuating headaches (Samsam et al., 2000). At the same time, vascular permeability changes, plasma components leak, platelets are activated, and meningeal mast cells undergo degranulation changes, resulting in aseptic inflammation and forming a vicious circle (Kilinc et al., 2020) (Irmak et al., 2019) (Kilinc et al., 2020). At present, the drugs for treating migraine include acetaminophen, nonsteroidal anti-inflammatory drugs, triptans, antiemetics, ergot alkaloids and combination analgesics, etc. But these drugs still cannot meet the needs of patients due to insufficient efficacy, obvious adverse reactions, and drug availability (Munro et al., 2017) (Kaube et al., 2002) (Gilmore and Michael 2011) (Huang Y et al., 2020). Even the recent development of monoclonal antibodies targeted to CGRP and its receptor also has potential cardiac liability issues (MaassenVanDenBrink et al., 2016). Therefore, there is an urgent need to find new treatments for migraine. More and more evidence show that botanical drugs or botanical drug preparations have broad prospects in the treatment of migraine. Such as feverfew (*Tanacetum parthenium* L.), butterbur (*Petasites hybridus* L.), marijuana (*Cannabis sativa* L.), *Ginkgo biloba* L. tree leaves, and *Ligusticum chuanxiong hort* etc. (Yun Luo et al., 2020) (Rajapakse and Davenport 2019) (Levin 2012) (D’Andrea et al., 2014).

**FIGURE 1:**
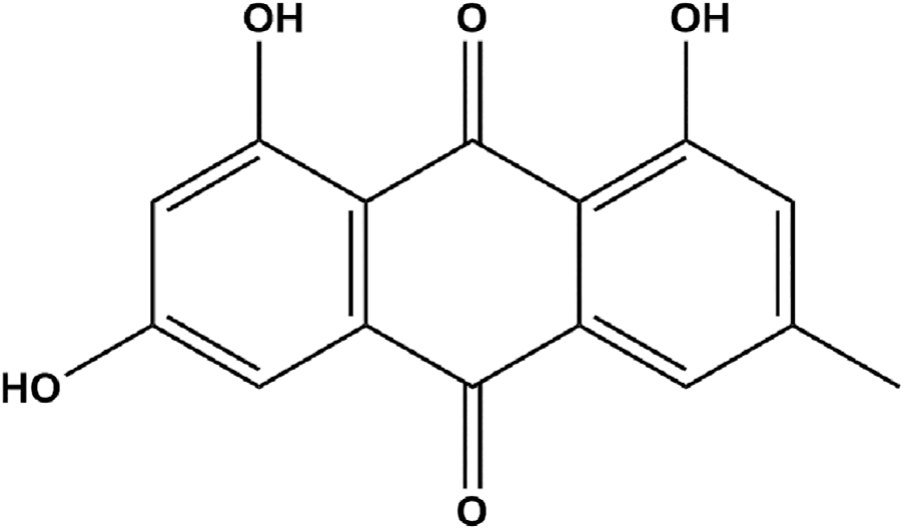
Chemical structure of emodin.

*Rheum officinale* Baill. (RO), recorded in the “XU Ming Yi Lei An” in the Qing Dynasty, which was processed with wine, and combined with tea to treat migraine. Emodin (Figure 1) is a plant-derived hydroxyanthraquinone, and can be separated from the traditional Chinese medicine RO. In recent years, a large number of studies on the pharmacological mechanism of emodin have found that emodin has the potential activity on cardiovascular diseases (Luo et al., 2021) and several nervous system diseases (Chen et al., 2021), with pharmacological effects in anti-inflammatory and contracting smooth muscle (Li et al., 2020). What’s more, emodin can reduce the release of CGRP in the trigeminal ganglion and inhibit orofacial pain (Xiong et al., 2017), but it is not clear whether emodin can relieve migraine caused by NTG. This research investigated the behavioral response of emodin to NTG-induced migraine rats; the levels of vasoactive factors NO, CGRP, SP, TNF-α and cGMP; the activity of c-Fos neurons and the expression of PKG protein.

## Materials and Methods

### Materials

98% pure emodin was purchased from Beijing Mindray Technology Co., Ltd. (batch no. M026282, Haidian district, Beijing, China). Ibuprofen granules were obtained from CSPC Group Co., Ltd. (batch no. 363200601, Shijiazhuang, Hebei, China). Sodium carboxymethyl cellulose (CMC-Na was purchased from Anhui Shanhe Pharmaceutical Excipients Co., Ltd. Company (batch no. 160511, Huainan, Anhui, China). Uratan was purchased from Sinopharm Chemical Reagent Co., Ltd. (batch no. 20190419, Shanghai, China). Nitroglycerin injection was purchased from Beijing Yimin Pharmaceutical Co., Ltd. (batch no. 20200310, Shunyi district, Beijing, China). Emodin and ibuprofen granules were respectively dissolved in 0.5% CMC-Na.

### Animals and Treatments

The adult SPF-grade Sprague-Dawley (SD) rats (48, half male and half female, weight range 200 ± 20 g) used in the study were provided by Hangzhou Medical College (Hangzhou, Zhejiang, China), and the animal approval number was SCXK (Zhejiang) 2019-0002. Rats were housed in plastic cages at 22 ± 2°C on a 12 h light/dark cycle with free access to food and water for 1 week. All animal experiments complied with ethical guidelines for the use of animals in research according to policies of the U.K. Animals (Scientific Procedures) Act, 1986 and associated guidelines, as well as with those of the Experimental Animal Administration of China Pharmaceutical University. What’s more, we had rationally explored and designed the dosage of the experiment to minimize the number of suffering rats.

After a week of adaptive feeding, 48 SD rats were randomly divided equally into four groups (*n* = 12): blank group (BG), model group (MG), ibuprofen group (IG) and emodin group (EG). The rats in BG and MG were undergone intragastric (i.g) injection of volume-matched 0.5% CMC-Na solution for 7 days before modeling, whereas the rats in IG and EG were administrated with 36 mg/kg of Ibuprofen and 27.3 mg/kg of Emodin respectively). The NTG injection was diluted to 10 mg/ml with 0.9% normal saline to obtain a white uniform emulsion, which was used to model migraine rats. After the half hour of the last i. g administration, all rats were subcutaneous (s.c) injected with GTN (10 mg/kg), excluding the BG. And the BG was s. c injected with a corresponding amount of sterile physiological saline solution. Because Kilinc et al. (2020) and Pradhan et al. (2014) in NTG-induced chronic migraine showed that no significant differences in between animals injected with 0.9% saline and the NTG solvent about the mechanical thresholds, CGRP content of plasma, trigeminal, ganglion, brainstem, and brain. Therefore, the vehicle control used in the experiments was 0.9% saline.

### Behavioral Research

Behavioral research was conducted in accordance with the previous literature. Briefly, the frequency of red ear symptoms, cage climbing and head scratching was observed and documented (Sun et al., 2017) (Hou et al., 2017). After model establishment, the animal showed symptoms of anxiety, accompanied with several regular symptoms, such as red ears, cage crawling, body shaking and head scratching for three to 5 minutes, which lasted for about 2 h. Among them, the pain symptoms of head scratching and cage climbing were relatively obvious, for which they were regarded as positive reactions of migraine. The figures of rats head scratching and cage climbing were observed and recorded continuously by observers who were blind to all treatment groups every 30 min for 2 h (*n* = 12).

### Biochemical Determination

After behavioral observation, all rats were sedated with 20% urethane (5 ml kg^−1^) by intraperitoneal injection. Blood samples were collected from abdominal aorta, gathered in centrifuge tubes and extemporaneously clotted for 30 min at room temperature. Then, they were centrifuged for 10 min at 3,000 rpm under a low temperature (4°C). The light-yellow supernatant was obtained which is serum. The level of NO in serum was detected according to the instructions of the nitric oxide biochemical kit (Nanjing Jiancheng Institute of Biological Engineering, Nanjing, China) by colorimetric method, while CGRP, SP, TNF-α and cGMP levels were determined with application of reagent test kits (CGRP, SP, TNF-α and cGMP kits: Nanjing Camilo Bioengineering Co., Ltd., Nanjing, China) according to the respective manual.

The enzyme-linked immunosorbent assay kit measures the content of CGRP, SP, TNF-α and cGMP in the serum. The protocol was carried out according to the manufacturer’s instructions. Briefly, 100 μL of sample or its standard was added to each well, while leaving a blank well. Then, the 96-well plates were incubated at 37°C for 90 min; later, the plate was washed twice, 100 μL of biotinylated detection Ab was added, and afterwards, the 96-well plates were incubated at 37°C for 60 min. After the incubation, wash the plate 3 times, except for the blank wells, 100 μL of HRP conjugate was added to each well and the plate was incubated at 37°C for 30 min. Afterwards, wash the plate 5 times, 100 μL of TMB working solution was added to each well, and the plate was incubated at 37°C for 15 min. After the incubation, instantly 100 μL of stop solution was added to each well. The optical density was measured at 450 nm using a microplate reader.

### Immunohistochemical Staining

After deep anesthetization, the hearts of rats were perfused with cold normal saline containing heparin sodium (0.9% sodium chloride +20 U/ml heparin sodium, 200-300 ml), until the rats’ livers turned white and bodies became rigid. Then use a scalpel to cut along the midline of the parietal bone of rats, then peel off the skin (scratched the middle of the parietal bone, but did not open it). After inserting the straight tweezer into the nostrils of rats, they broke to both sides, and then the parietal bone cracked. Carefully remove the whole brain with the curved forcep. Carefully remove the whole brain with the curved forcep. Half of the brain tissue was fixed with 4% paraformaldehyde solution for 24 h for immunohistochemistry research (the remaining half of the brain tissue was stored in a −80°C degree refrigerator for Western blot research). Subsequently, the brains were dehydrated with 20% sucrose solution and embedded in tissue freezing medium (OTC). Then the brain tissues were cut into slices of uniform thickness 4 μm. (*n* = 6, half coronal cut, half sagittal cut). The specific operation of immunohistochemistry was completed according to a previous study (X. F. Zhang et al., 2017).

Further, the brain sections were observed using a microscope with 200× of magnification. The positive c-Fos neurons were brown-yellow, while the negative neurons showed blue-purple. Ultimately, the Image-Pro Plus (U.S. MEDIA CYBERNETICS) software was used to count the number of c-Fos positive cells and analyze the integrated optical density (IOD) of positive staining.

### Western Blot

The brain tissues were pulverized with mortars in liquid nitrogen. About 100 mg of brain tissue powder was weighed and the proteins were extracted with 1 ml of RIPA Lysis Buffer which contained 10 μL of PMSF. Then the quantitation of proteins was conducted with a BCA protein concentration determination kit. After protein quantification, the protein samples were stored at −80°C for later use. According to the literature (Shi et al., 2020) (Ong et al., 2002), for PKG1 protein, the total protein was separated on 10% gradient sodium dodecyl sulfate-polyacrylamide gel electrophoresis (SDS-PAGE) at 100 V and then transferred onto polyvinylidene fluoride (PVDF) membranes (Millipore, United States). Afterwards, the membranes were blocked with 10% skim milk for 2 h at room temperature and then incubated with primary antibodies including GAPDH (1:1000; cell signaling technology; catalog number; 2118), PKG1 (1:1000; Proteintech; catalog number: 21646-1-AP) overnight. The next day, the membranes were washed with TBST solution three times (10 min per time), following 2 h of incubation with secondary antibodies (1:4,000, Hangzhou Fude Biological Technology Co., LTD, catalog number: 3256751) at room temperature. For visualization, the ECL was applied for the color development of protein bands. The protein intensity was measured with Image-Pro Plus 6.0 software. Data are expressed as ratios of the PKG1/GAPDH.

### Data Analysis

All data were presented as mean ± SD and analyzed by a one-way analysis of variance, followed by Tukey’s multiple comparisons as post hoc test. All statistical analyses and graphs were performed using GraphPad Prism version 7.0 software (GraphPad Software Inc., San Diego, CA, United States). *p* values less than 0.05 were considered statistically significant.

## Results

### Emodin Diminished the Number of Head Scratching and Cage Climbing of NTG-Induced Migraine Rats

**FIGURE 2:**
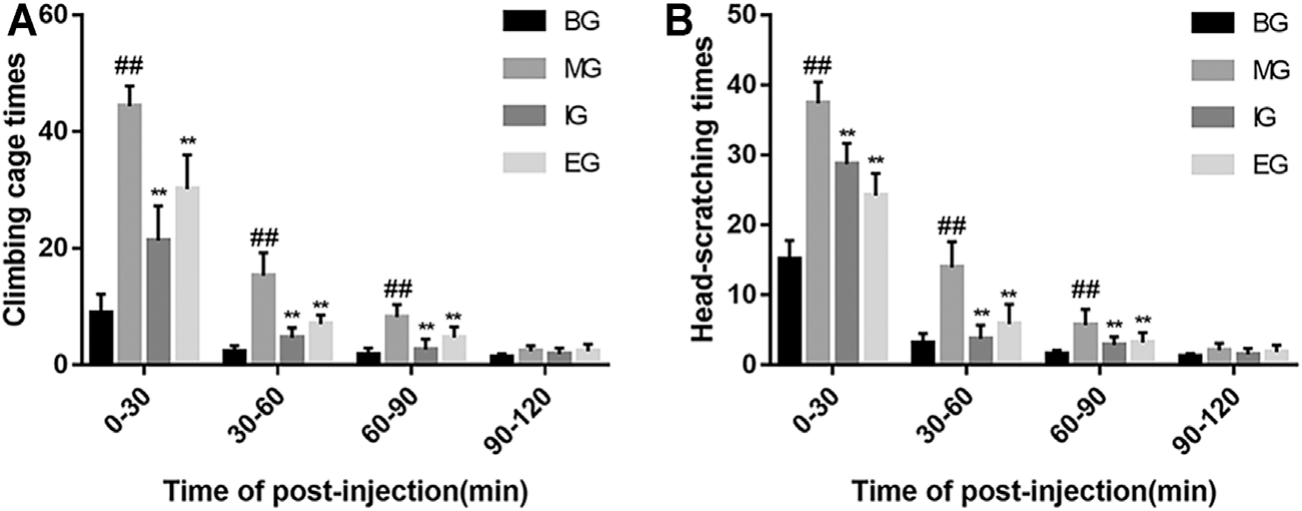
Pain response of rats in different groups after NTG treatment. **(A)** The number of cages climbing in each rat group. **(B)** The number of head scratching in each rat group. Within 2 h after the injection of nitroglycerin, the number of cages climbing and head scratching were respectively recorded every 30 min. Data were expressed as mean ± SD (*n* = 12); ^##^ *p* < 0.01 vs. the BG; ^**^ *p* < 0.01 vs. the MG. Emodin reduced NO levels after NTG treatment.

Figure 2 showed that rats in the BG displayed a small amount of head scratching and cage climbing times during the observation period. Within 30 min after treatment of NTG, the frequency of both head scratching and cage climbing behaviors of rats in the MG and the drug interference groups were markedly increased, while within 30–90 min, the frequency of these behaviors showed varying degrees of decline. Compared with the BG rats, the number of head scratching and cage climbing were notably increased than that in MG (*p* < 0.01) between 0 and 90 min after NTG treatment. However, the numbers of head scratching and cage climbing of the drug interference groups rats were considerably lower than that in the MG 0–90 min after NTG treatment (*p* < 0.01).

**FIGURE 3:**
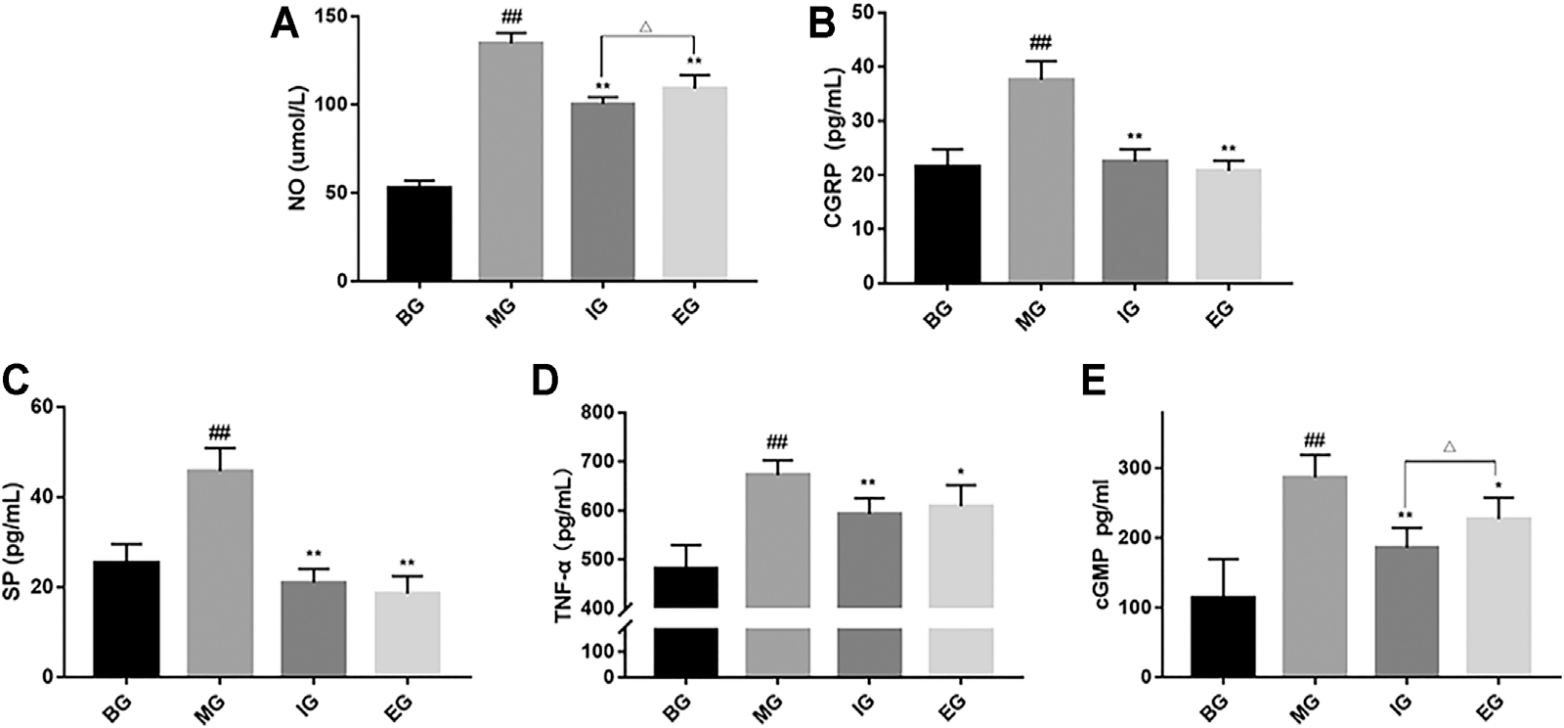
Effect of emodin on vasoactive substances in the serum after NTG treatment. **(A)** Nitric oxide (NO), **(B)** Calcitonin gene-related peptide (CGRP), **(C)** substance P (SP), **(D)** TNF-α and **(E)** cGMP. Data were presented as mean ± SD (*n* = 6), ^##^ *p* < 0.01 compared with the BG; *p < 0.05, **p < 0.01, compared with the MG; ^Δ^ *p* < 0.05, compared with the IG.

As shown in Figure 3A, compared with the BG, the MG significantly increased the level of NO in the body (*p* < 0.01). Compared with the MG, ibuprofen granules and emodin can significantly reduce the level of NO (*p* < 0.01). Moreover, the inhibitory effect of the IG was stronger than that of the MG (*p* < 0.05), which is consistent with the results of behavioral studies.

### Emodin Reduced CGRP Levels After NTG Treatment

It can be seen from Figure 3B that compared with the BG, the CGRP level of the MG was significantly higher than that of blank rats (*p* < 0.01). Pretreatment with ibuprofen and emodin could significantly reverse the increase in CGRP levels (*p* < 0.01), and there is no significant difference between the IG and the MG (*p* > 0.05).

### Emodin Reduced SP Levels After NTG Treatment

As shown in Figure 3C, compared with the BG, the level of SP in the MG was significantly higher than that in the BG (*p* < 0.01), and SP levels in the serum of group ibuprofen and emodin rats showed no significant difference (*p* > 0.05). Compared with the MG, the IG and the EG can significantly inhibit the increase in SP content (*p* < 0.01).

### Emodin Reduced TNF-α Levels After NTG Treatment

The results were shown in Figure 3D, compared with the BG, NTG injection increased the levels of TNF-α in serum of model rats (*p* < 0.01). However, compared with the MG, the IG and the EG could significantly inhibit the increase of TNF-α level (*p* < 0.05).

### Emodin Reduced cGMP Levels After NTG Treatment

It can be seen from Figure 3E, compared with the BG, NTG injection increased the levels of cGMP in the serum of model rats (*p* < 0.01). However, compared with the MG, the IG and the EG could significantly inhibit the increase of cGMP level (*p* < 0.05). Moreover, the inhibitory effect of the IG was stronger than that of the MG (*p* < 0.05).

### NTG-Induced Activation of c-Fos Immunoreactive Neurons

**FIGURE 4:**
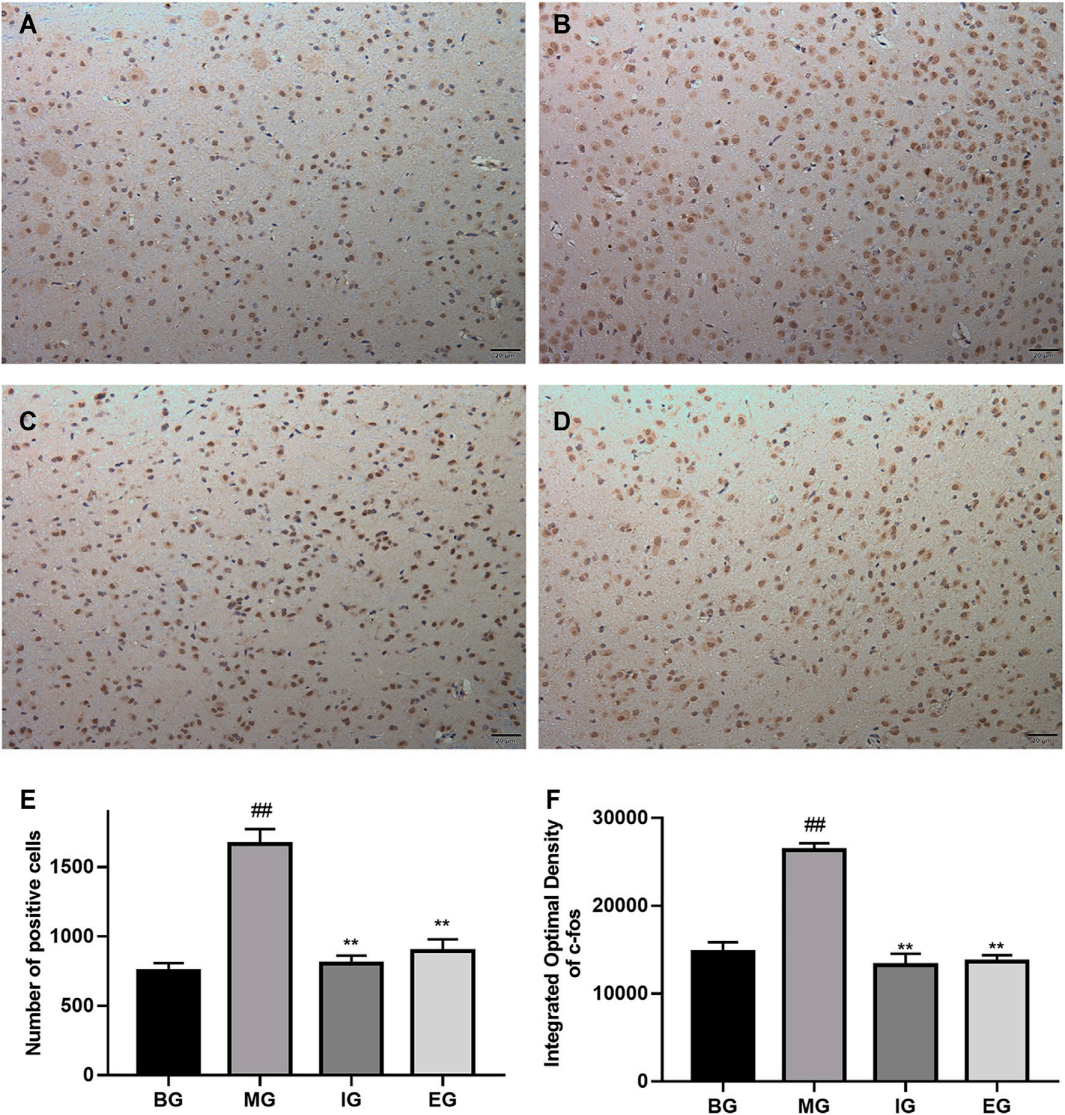
The result of emodin on c-Fos neurons in the cerebral cortex after NTG treatment (coronal cut). Immunohistochemistry photos: **(A)** BG, **(B)** MG, **(C)** IG, **(D)** EG; bar graph: **(E)** the number of c-Fos positive cells; **(F)** Integral Optical Density (IOD); photos were magnified 200 times: scale bar = 20 μm; data was expressed as mean ± SD (*n* = 6), ^##^ *p* < 0.01 compared with the BG; **p < 0.01, compared with the MG.

**FIGURE 5:**
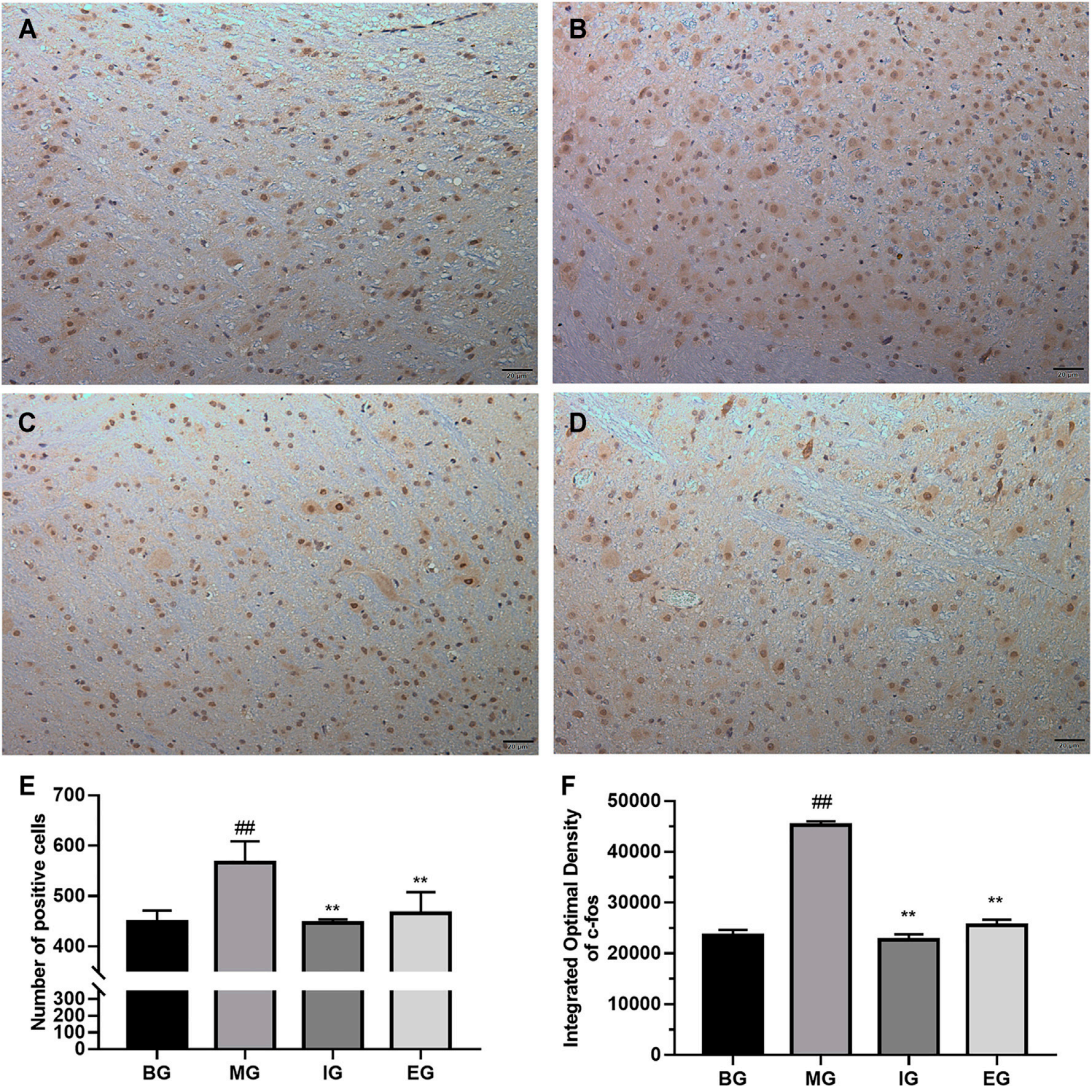
The response of emodin on c-Fos neurons in the brainstem after NTG stimulation (sagittal cut). Immunohistochemistry photos: **(A)** BG, **(B)** MG, **(C)** IG, **(D)** EG; bar graph: **(E)** the number of c-Fos positive cells; **(F)** Integral Optical Density (IOD); taken photos under microscope at 200× magnification: Scale bar = 20 μm; values were represented as the mean ± SD (*n* = 5). ^##^ *p* < 0.01 vs. the blank, **p < 0.01 vs. the model.

We studied the effect of emodin on the activation of c-Fos neurons. The expression of c-Fos in different brain tissue regions (cortex, hippocampus, cerebellum, brainstem) was detected by immunohistochemistry. In addition, it was found that the difference in c-Fos expression between different groups was mainly reflected in the cortex and brainstem. The results were shown in Figures 4, 5.

NTG-activated c-Fos immunoreactive neurons were brownish yellow, while negative cells were blue purple. Obviously, there existed a lot of brown-yellow clumps in Figure 4B than that in Figure 4A. And the brown-yellow positive expression in Figures 4C,D was significantly reduced than that in Figure 4B. It can be seen from Figure 4E that the number of c-Fos positive expressing cells in the MG was greater than that in the BG (*p* < 0.01). In contrast, ibuprofen and emodin pretreatments significantly down-regulated the number of c-Fos positive cells (*p* < 0.01). As shown in Figure 4F, compared with the BG, the IOD of the MG was much larger (*p* < 0.01), while ibuprofen and emodin greatly deceased the IOD of c-Fos positive cells (*p* < 0.01). All of which indicated that pretreatment with ibuprofen and emodin can reduce the overexpression of c-Fos neurons in the cortex.

Similarly, it was found from Figure 5E that the number of c-Fos positive cells in model rats was larger than that in blank rats (*p* < 0.01), and pretreatment with ibuprofen and emodin could reverse this increase (*p* < 0.01). As shown in Figure 5F that compared with the BG, the IOD of the stained area in the MG was larger (*p* < 0.01), but after ibuprofen and emodin pretreatment, the IOD of the stained area was significantly reduced (*p* < 0.01), there was no significant statistical difference between the IOD of the IG and the EG (*p* > 0.05). The results were consistent with those shown in Figures 5A–D. In Figure 5B, there were numerous brown-yellow positive cell areas, while in Figure 5A, there was a bit of brown-yellow positive cell areas in the stained sections of the BG. The brownish yellow area in Figures 5C,D was much smaller than the brown-yellow area in Figure 5B, which indicated that pretreatment with ibuprofen and emodin could reduce the overexpression of c-Fos neurons in the brainstem.

### Effects of NTG-Induced on the Expression Levels of PKG

**FIGURE 6:**
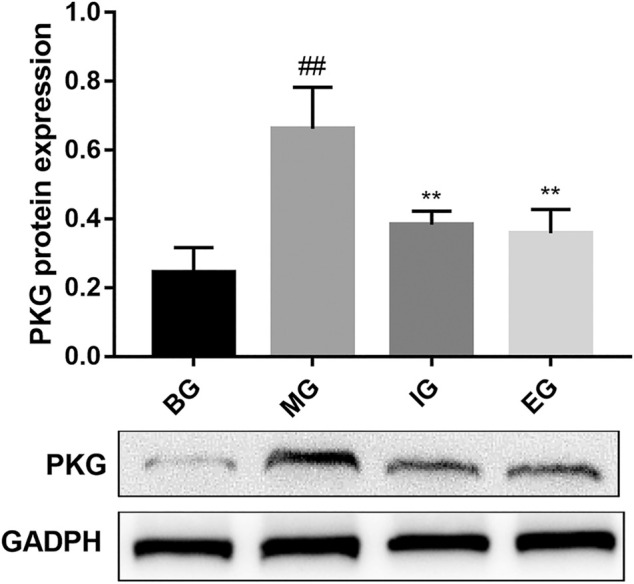
The effect of emodin on the level of PKG protein in brain tissue induced by NTG. Values were represented as the mean ± SD (*n* = 6). ^##^ *p* < 0.01 versus normal, and **p < 0.01 *versus* the model.

In order to study the effect of emodin on the PKG protein induced by NTG, western blot analysis was performed on the brain tissue protein. As shown in Figure 6, compared with the BG, the expression of PKG protein in the MG increased significantly (*p* < 0.01); compared with the MG, ibuprofen and emodin could significantly inhibit the increase of PKG protein (*p* < 0.01), and there was no significant statistical difference in the inhibitory intensity between the two groups.

## Discussion

Migraine is a serious neurological disease which is frequent but tends to be neglected. Due to the insufficient diagnosis rate, the high misdiagnosis rate and the lack of medication, many patients are still suffering from the disease silently (Viana et al., 2020). In this study, we demonstrated that emodin (27.3 mg kg^−1^) can relieve migraine-like headaches through the vascular nervous system. Subcutaneous injection of NTG can activate the NO-cGMP pathway to cause migraine (Thomsen et al., 1994). The symptoms of migraine include head scratching, cage climbing, tremor, ear redness, irritability, etc. (Hou et al., 2017; Zhang et al., 2017). Behavioral results showed that after subcutaneous injection of NTG, migraine rats seemed to be more active, and after emodin was given, the number of head scratching and cage climbing within 90 min was significantly reduced. Therefore, this research showed that emodin could improve the abnormal behavior of migraine.

In this study, it was found from Figure 3 that after subcutaneous injection of NTG, the levels of NO, CGRP, SP, TNF-α and cGMP in rats increased, but emodin could significantly reduce the levels of NO, CGRP, SP, TNF-α and cGMP in rats, which showed that emodin has a potential anti-migraine activity. Intravenous infusion of NTG (NO donor) can activate the trigeminal nerve vascular system and cause migraine (Capuano et al., 2014). The modeling mechanism is that NO activates s-GC in smooth muscle to cause cells to generate a large amount of cGMP (Tassorelli et al., 2004; Olesen 2008) thereby activating cGMP-dependent proteases PKG (Stepieni 1998). The proteases PKG reduces the binding of contractile protein to Ca^2+^ and leads to excessive vasodilation, leading to plasma protein extravasation or degranulation of meningeal mast cells, resulting in aseptic inflammation, and increased levels of inflammatory factors such as TNF-α, IL-6, and IL-1β. In addition, during the onset of migraine, the release levels of vasoactive peptide CGRP and SP are also significantly increased (May and Goadsby 2001; Farajdokht et al., 2018). Therefore, vasoactive factors such as NO, CGRP, SP, TNF-α and cGMP can be used as important biochemical indicators for the diagnosis of migraine. However, Ebersberger et al. (1999) and Kilinc, et al. (2020) reported that neurogenic inflammation did not change SP levels. We believe the discrepancy could be explained by the different model methods they used. Moreover, Luo et al. (2020), Wen et al. (2019), and Shao et al. (2013) used subcutaneous injection of NTG (10 mg/kg) to model migraine. Compared with the blank group, the substance P in the model group was significantly increased, which is consistent with the result we got.

Previous studies showed that subcutaneous injection of NTG could promote the expression of c-Fos (Bolay 2012) (Barbanti et al., 2020) (Bohár et al., 2013) (Y. Zhang et al., 2020). As in our study, c-Fos neurons were highly expressed in the brainstem and the cortex of migraine rats. And emodin pretreatment reduced the number of c-Fos positive cells, the size of the staining area and the IOD value in NTG-treated cerebral cortex and brainstem of rats. So, it was proved that emodin reduced the overactivity of c-Fos neurons and played an important role in the therapy of migraine. As we all know, preclinical studies related to migraine commonly use trigeminal nucleus caudalis in the brainstem where pain signals are transmitted to second-order neurons to determine c-Fos activation (Ramachandran et al., 2012) (Noghani et al., 2019) (Zhu et al., 2011) (Offenhauser et al., 2005). But in our research, the c-Fos of rat brain cortex treated by NTG was also overexpressed, and the possible reason is that higher pain centers are eventually activated all the way to the sensory cortex, where headache is finally perceived (Ramachandran et al., 2012).

**FIGURE 7:**
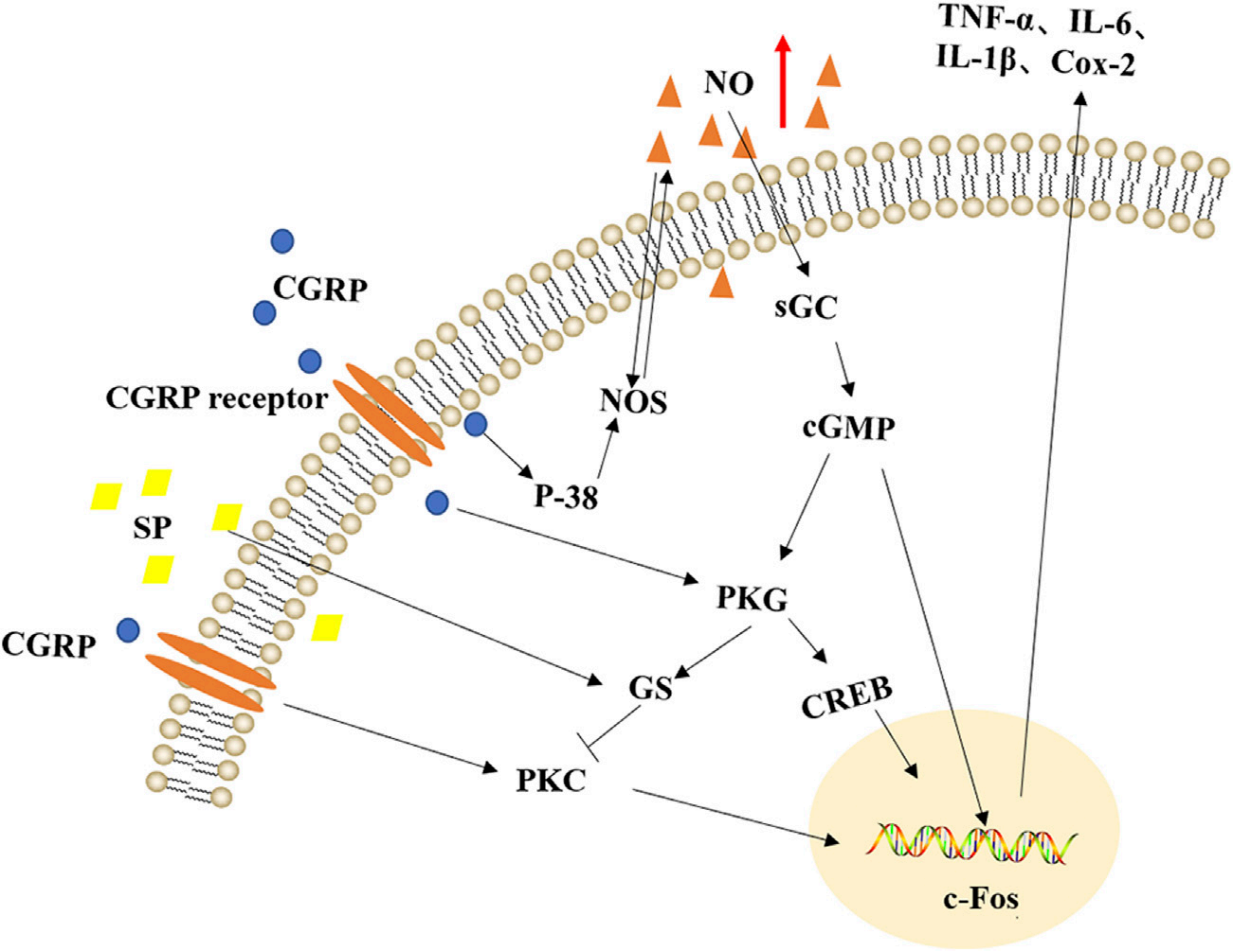
The mechanism roadmap of nitroglycerin-induced migraine rats. NO, nitric oxide; SP, substance P; CGRP, calcitonin gene-related peptide; PKC, protein kinase C; cGMP, cyclic guanosine monophosphate; PKG, protein kinase G; s-GC, Soluble guanylyl cyclase; GS, (RefSeq) G protein subunit alpha L; CREB, cyclic adenosine monophosphate response element binding factor.

In this study, we discovered that emodin could inhibit the expression of PKG protein in migraine rats. The best-known function of PKG is to promote the relaxation of vascular smooth muscles and cause arterial vasodilation (Qi et al., 2007). The physiological effect of NO depends on the cGMP-dependent signal conversion pathway, and cGMP is the key to the second messenger in the cell, which is the main influencer of PKG (Ren et al., 2020). What’s more, some researchers compared the genome differences between people with chronic generalized pain and healthy subjects and found PRKG1 genes play a role in patients with chronic generalized pain effect (Polli et al., 2020). Therefore, inhibiting the expression of PKG protein can reduce the dilation of cerebral blood vessels, and relieve migraine. In addition, as shown in Figure 7, in model rats, NTG (NO donor) could cause vascular endothelial cell depolarization, cerebral vasodilation, and then caused aseptic inflammation, such as increased release of TNF-α and IL-6; at the same time, it activated the NO-cGMP-PKG pathway, the content of SP, CGRP and cGMP were increased, which led to migraine. Emodin could treat migraine by inhibiting the production of NO in migraine rats, the release of TNF-α inflammatory factors, the production of vasoactive peptide SP, CGRP, and the expression of PKG protein.

All experimental results demonstrated that emodin could play an anti-migraine role, and the specific reasons are: 1) Emodin can inhibit NO generation, reduce the excessive expansion of blood vessels. 2) Emodin reduces the release of inflammatory factor TNF-α. 3) Emodin can reduce the release of vascular neuropeptides CGRP and SP. 4) Emodin can inhibit the expression of PKG protein in migraine rats and reduce vasodilation. All of these indicate the possibility of emodin as an anti-migraine drug.

## Conclusion

This study indicated that emodin could alleviate NTG-induced migraines in rats. The therapeutic effect was attributed to anti-inflammatory and inhibiting vasodilation. And the mechanism was that emodin could reduce the expression of PKG protein in rat brain tissue induced by NTG through the cGMP-PKG pathway and weaken the activation of c-Fos. This research has a great significance to the development of anti-migraine drugs.

### Limitations of the Study

As we all know, female migraine sufferers are 3 times as common as males, but its underlying mechanisms remain unclear. In a recent study, Cetinkaya et al. (2020) investigated the effects of female sex hormones estrogen and progesterone on CGRP and SP in *in-vivo* and *ex-vivo* in rats of both sexes. They found that progesterone increased both plasma levels of CGRP and SP. Also, estrogen did not change the basal CGRP levels in the plasma in both intact female and ovariectomized rats. But in the present study, we chose half of the male rats and half of the female rats to detect the levels of CGRP and SP factors in the serum. Therefore, it is not thoughtful to join female rats for research. In future studies, we will only use male rats or compare the differences between rats of different sexes for migraine research.

Pain, as defined by the International Association for the Study of Pain (IASP), is “an unpleasant sensory and emotional experience associated with actual or potential tissue damage, or described in terms of such damage.” It cannot be directly measured in animals; instead, pain is inferred from “pain-like” behaviors. Pain and behavioral outcomes are challenging to assess in preclinical rodent studies. Most studies have tried to indirectly assess pain by non-evoked behaviors. Currently, mechanical or thermal hyperalgesia tests are used widely to measure pain migraine models in rodents (Deuis, Dvorakova, and Vetter 2017; Vuralli et al., 2019). In addition, Von Frey monofilaments and Electronic von Frey are quick and reliable quantitative sensory testing to explore mechanical hyperalgesia with positive reproducibility sex (Tena et al., 2012). But we only observed the behavior of crawling and scratching the head of migraine rats. If the von-Frey test is added to the behavioral study, it will have a great advantage.

## Data Availability

The original contributions presented in the study are included in the article/Supplementary Material, further inquiries can be directed to the corresponding authors.
