# Supplementary material for: Emodin Interferes With Nitroglycerin-Induced Migraine in Rats Through CGMP-PKG Pathway
Source: Front Pharmacol. 2021 Oct 20;12:758026. doi: 10.3389/fphar.2021.758026 (PMC8563583; doi:10.3389/fphar.2021.758026)
Supplement: Supplementary file 1 [file DataSheet2.docx]

**Supplementary file 2**


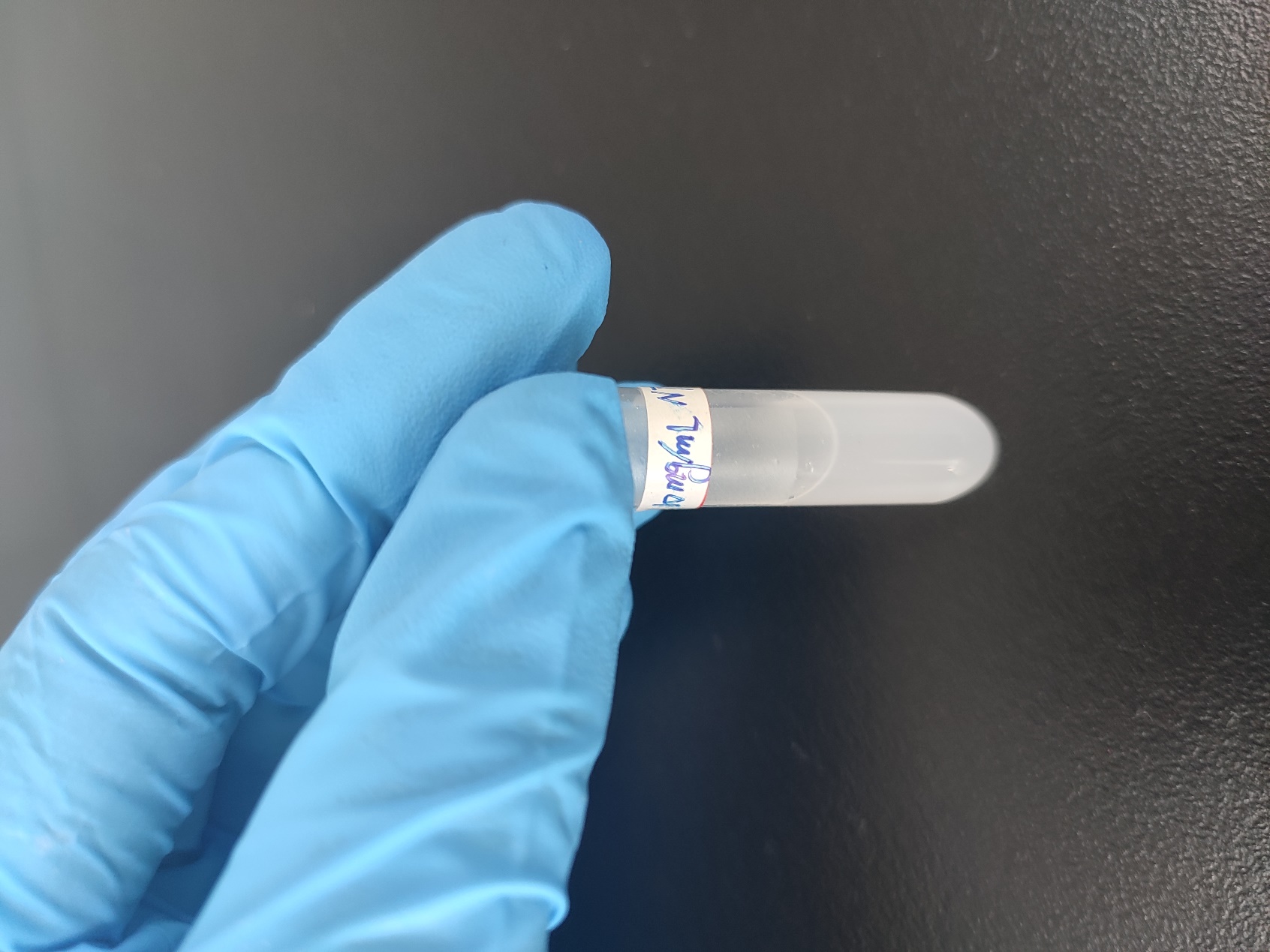


Figure 1. Nitroglycerin Modeling Fluid


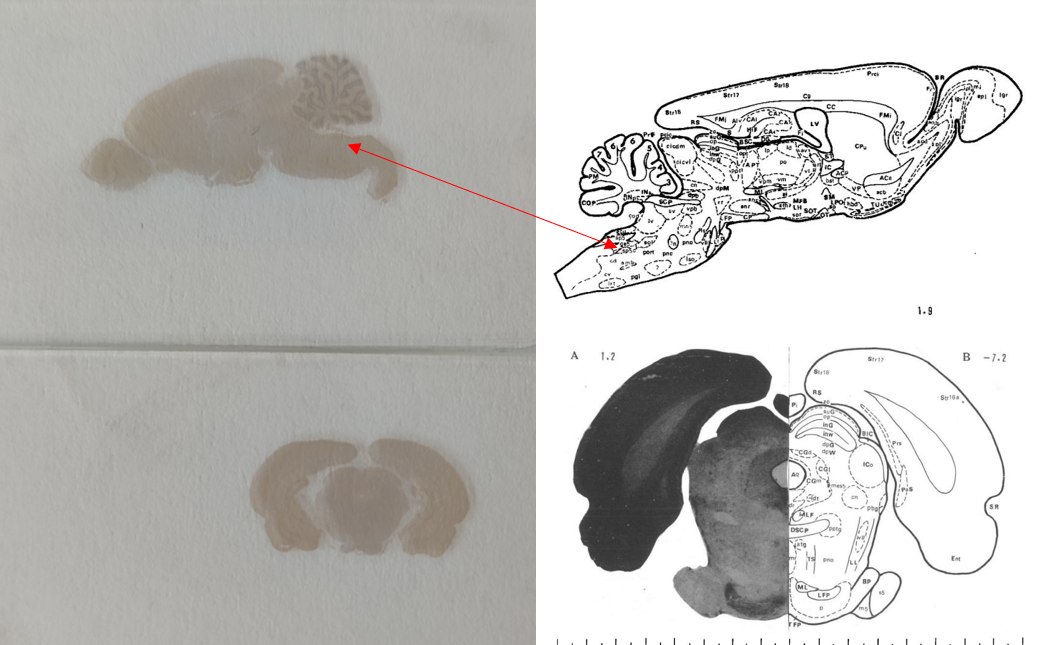


Figure 2. c-Fos immunohistochemistry brain slice


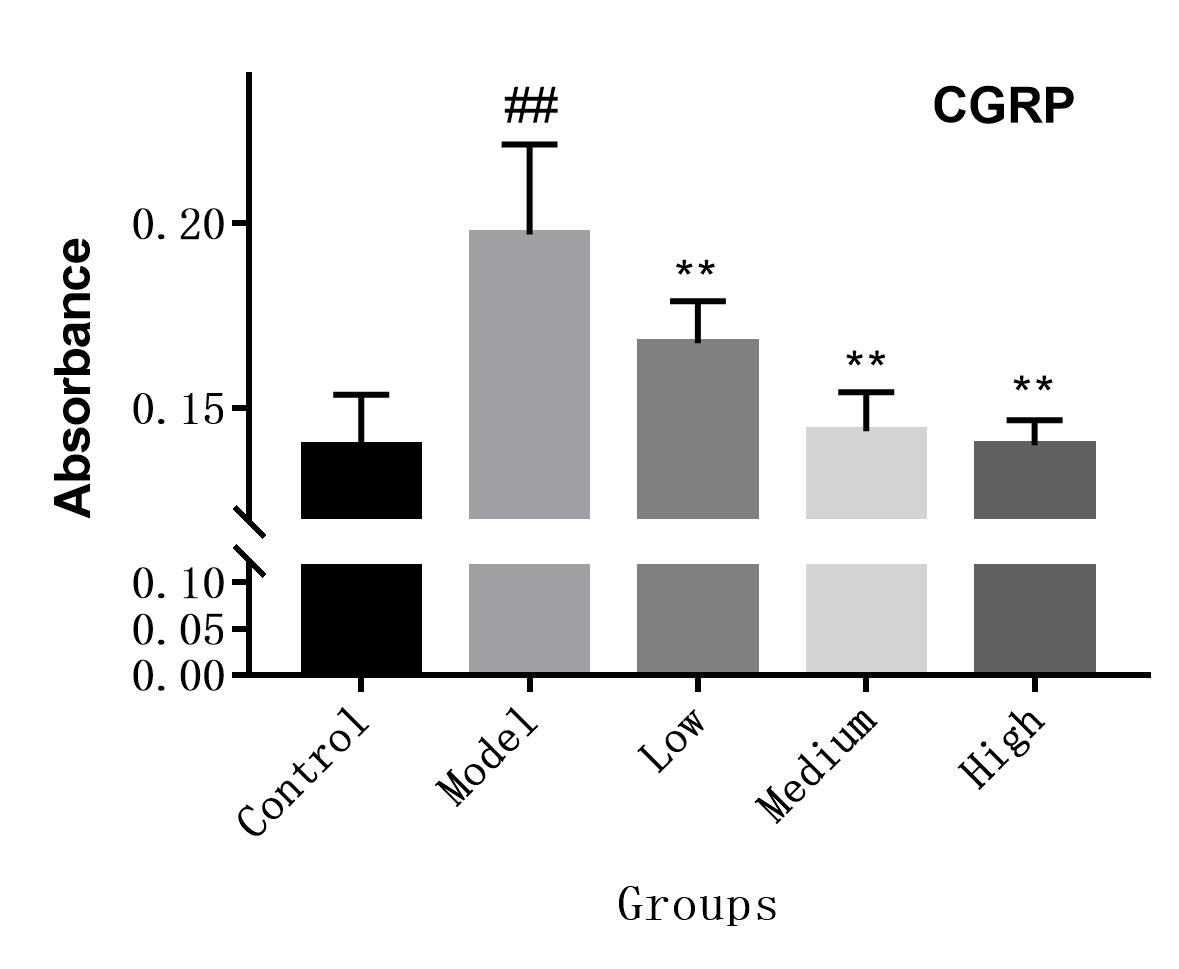


Figure 3. The effect of *Rheum officinale* Baill. on CGRP in rat serum induced by NTG
