# Supplementary material for: Emodin Interferes With Nitroglycerin-Induced Migraine in Rats Through CGMP-PKG Pathway
Source: Front Pharmacol. 2021 Oct 20;12:758026. doi: 10.3389/fphar.2021.758026 (PMC8563583; doi:10.3389/fphar.2021.758026)
Supplement: Supplementary file 2 [file DataSheet1.docx]

**Supplementary file 1**


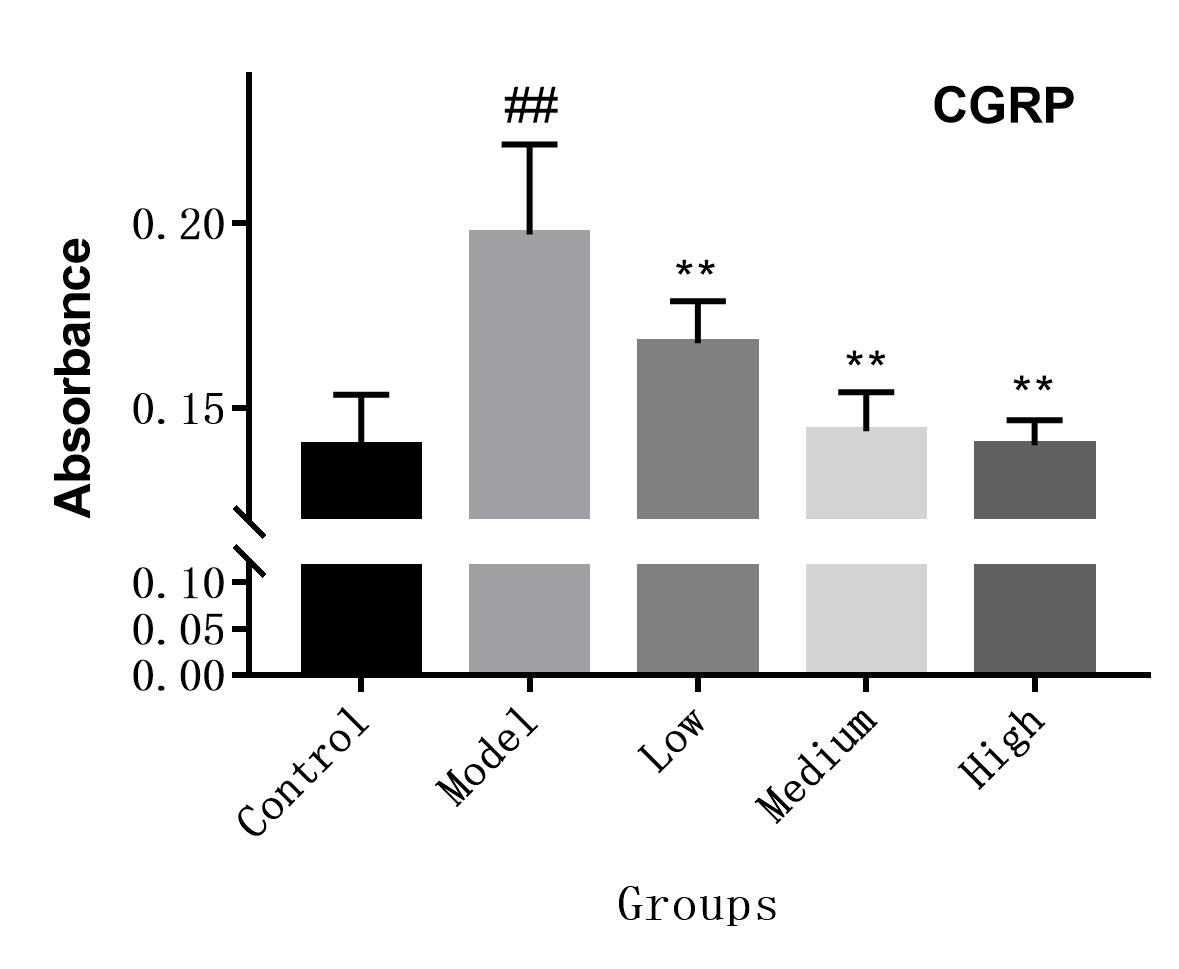


Figure 1. The effect of *Rheum officinale* Baill. on CGRP in rat serum induced by NTG


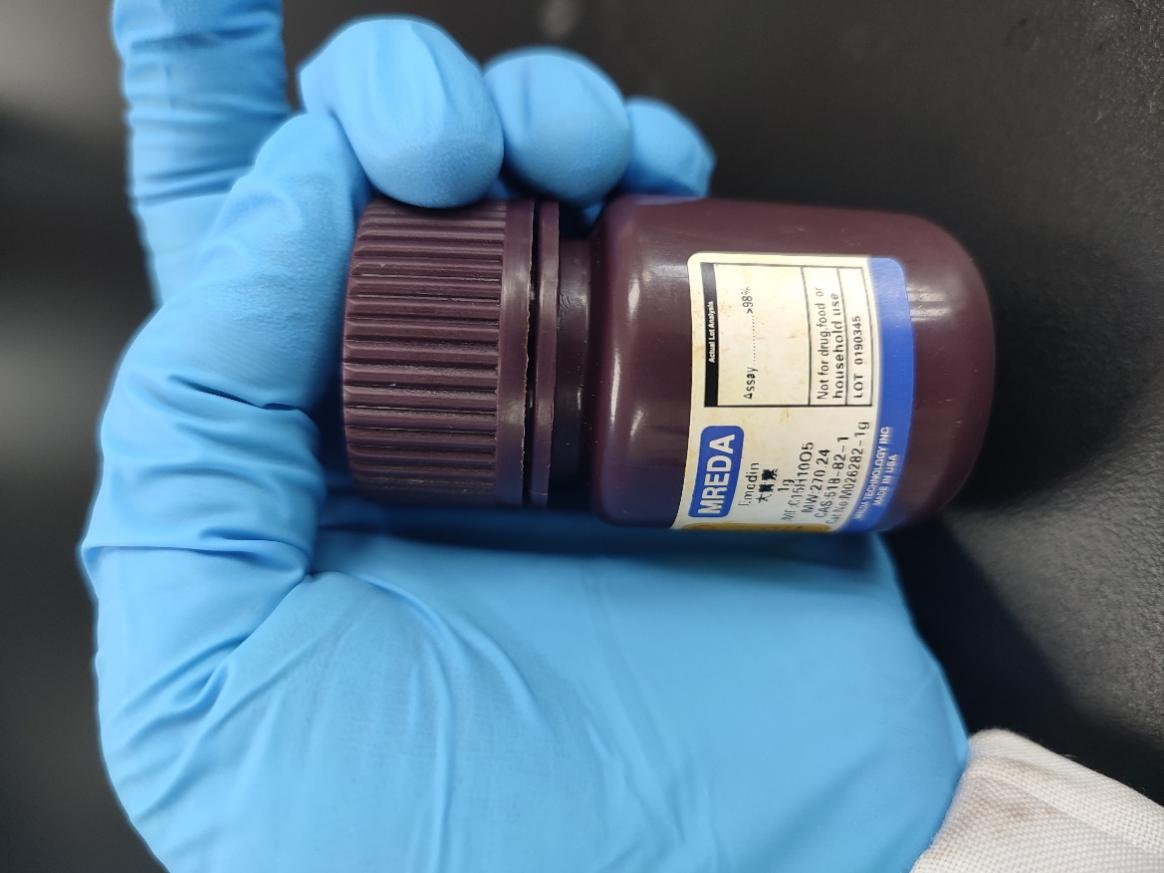


Figure 2. Bottled emodin


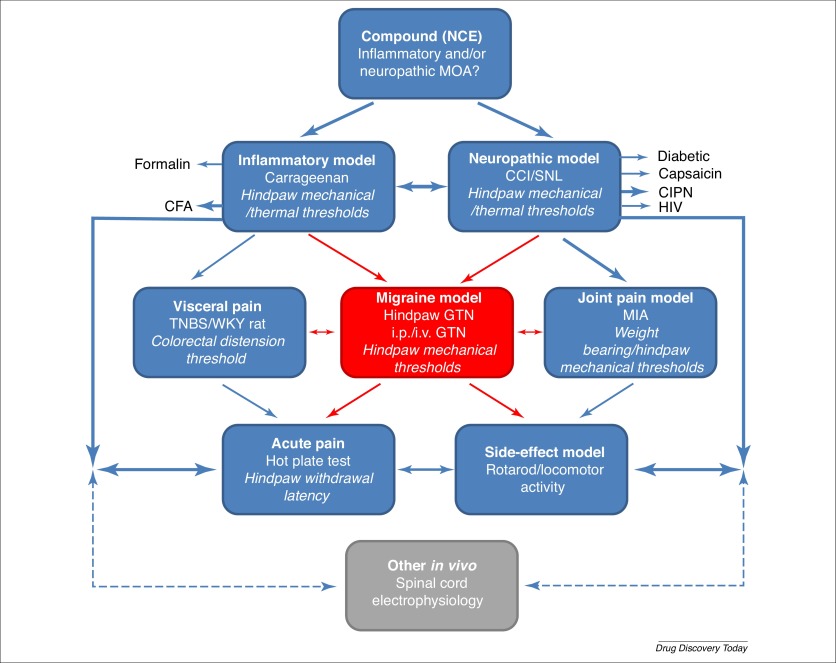


Figure 3. Typical in vivo platform for use in pain drug discovery testing(Munro, Jansen-Olesen, and Olesen 2017).
